# Genetic Mapping of Anaerobic Germination-Associated QTLs Controlling Coleoptile Elongation in Rice

**DOI:** 10.1186/s12284-015-0072-3

**Published:** 2015-12-23

**Authors:** Sheng-Kai Hsu, Chih-Wei Tung

**Affiliations:** Department of Agronomy, National Taiwan University, No. 1, Sec. 4, Roosevelt Rd, Taipei, 10617 Taiwan

**Keywords:** Rice, Quantitative Trait Loci, Genome-Wide Association Study, Anaerobic Germination, Coleoptile Elongation, Direct Seeding

## Abstract

**Background:**

Increasing numbers of rice farmers are adopting direct-seeding methods to save on costs associated with labor and transplanting. Successful seedling establishment in flooded conditions requires rapid coleoptile growth to ensure access to oxygen near the water surface. It is important that the natural variations in coleoptile growth of submerged rice plants are identified.

**Results:**

Coleoptile responses of submerged plants at the germination stage were analyzed in diverse rice accessions and recombinant inbred lines. Several genomic regions identified from a genome-wide association analysis were significantly associated with anaerobic germination, with many that corresponded to published quantitative trait locus (QTL) intervals. In the recombinant inbred line population derived from a cross between *japonica* and *indica* varieties, only one unique and strong signal explaining about 27 % of the phenotypic variation was detected. Distinct haplotypes associated with variations in coleoptile length were identified in diverse germplasm.

**Conclusions:**

We demonstrated the value of combining genome-wide association analysis and biparental QTL mapping approaches to identify chromosomal regions regulating coleoptile elongation in submerged rice plants. The significant genomic regions detected in this study are potential candidates for incorporation into elite cultivars to improve seedling survival during anaerobic germination. Future studies that map the QTLs and investigate the effects and functions of candidate genes may lead to new rice varieties that can be used in direct-seeding systems.

**Electronic supplementary material:**

The online version of this article (doi:10.1186/s12284-015-0072-3) contains supplementary material, which is available to authorized users.

## Background

Direct seeding is becoming a popular method in some rice-growing areas, especially in Southeast Asian countries, because of its low cost and convenience. However, poor seedling establishment has been a major obstacle preventing the large-scale adoption of direct-seeding methods. Many environmental and genetic factors affect early rice growth and establishment in the field. For example, flooding due to rainfall or uneven agricultural land creates unfavorable conditions for rice to germinate and develop strong and uniform seedlings. Therefore, breeding rice varieties capable of surviving under flooded conditions during germination and early growth stages will improve the success of direct-seeding cultivation (Pandey et al. 2002).

Unlike most cereal crops, rice is able to germinate and grow in semi-aquatic environments. When germinating while submerged in water, rice seeds are exposed to oxygen deficiency and limited sources of energy. Therefore, to acquire enough oxygen to fuel required metabolic activities, the coleoptile elongates rapidly after germination. The faster the coleoptile elongates, the sooner the seedling is able to avoid flooding stress, which improves the chances the plant will survive. However, not all rice varieties can tolerate germination in submerged conditions. Unique anaerobic germination processes may be more efficient in some rice genotypes (Miro & Ismail 2013).

Rice is unique in its ability to anaerobically mobilize the starchy reserve in the endosperm, which satisfies the metabolic requirements of the non-photosynthetic embryonic axis during anaerobic germination (Guglielminetti et al. 1995a, 1995b; Hwang et al. 1999; Perata et al. 1997). Rice α-amylase is a critical enzyme in the mobilization of the starchy reserve by degrading intact starch granules into glucose polymers, which are digested into soluble sugars by hydrolase. A whole genome transcriptome analysis has been conducted to identify differentially expressed genes in rice coleoptiles in anoxic conditions (Lasanthi-Kudahettige et al. 2007). They observed that *RAmy3D* is highly induced, indicating that it may be active during anaerobic mobilization of rice. Additionally, their results suggested that genes involved in glycolysis and anaerobic fermentation were up-regulated during exposure to anoxic conditions to maintain a sustainable energy supply for survival. An oxygen/energy-deficit sensing mechanism was proposed based on studies involving mutants. The *calcineurin-interacting protein kinase* (*CIPK15*) gene was reported to play an important role in a signaling pathway that regulated *RAmy3D* expression, which affected the anaerobic germination of rice (Lee et al. 2009; Lu et al. 2002; Lu et al. 2007). Studies of tolerant and intolerant genotypes showed higher amylase activity and *RAmy3D* expression in tolerant varieties (Ismail et al. 2009; Magneschi et al. 2009). However, it is still unclear whether gene sequence variations are associated with the ability to germinate anaerobically in diverse rice varieties.

In recent years, rice scientists conducted a series of linkage analyses using several biparental mapping populations to identify the quantitative trait loci (QTLs) associated with tolerance to flooding during anaerobic germination. Shoot lengths of 81 recombinant inbred lines (RILs) derived from the Kinmaze/DV85 cross and 148 F_2_ progenies of the USSR5/N22 cross grown under anoxic conditions were measured for QTL analyses. Seven QTLs on chromosomes one, two, five, seven, and 11 were detected (Jiang et al. 2004; Jiang et al. 2006). Research groups at the International Rice Research Institute (IRRI) screened several mapping populations for seedling germination and survival rate under submerged conditions and identified several QTLs associated with anaerobic germination (Angaji et al. 2010; Baltazar et al. 2014; Septiningsih et al. 2013). These QTLs have been targeted for molecular cloning and breeding (Miro & Ismail 2013). The first natural variant of QTL *qAG-9-2* enhances anaerobic germination and was recently fine-mapped to *OsTPP7*, which is a gene encoding a trehalose-6-phosphate phosphatase (Kretzschmar et al. 2015). Functional characterization of *OsTPP7* suggested its involvement in enhancing starch mobilization to drive embryo germination and coleoptile elongation.

Many factors contribute to the success of seedling establishment in flooded soil. In the late 1990s, researchers at the IRRI completed a series of pioneering studies to investigate how seed sowing conditions, cultivar genotypes, seedling physiological characteristics, and seed vigor affected the germination and development of seedlings in hypoxic conditions (Yamauchi et al. 1993; Yamauchi et al. 1994; Yamauchi & Chuong 1995; Yamauchi & Biswas 1997). Their research proved that coleoptiles of flooding-tolerant cultivars grew faster and longer in flooded soil. This morphological adjustment enabled the superior cultivars to access oxygen at the water surface earlier, which supported the growth of organs such as the primary leaf and root. This ultimately ensured the survival of the seedling.

In this study, we used genome-wide association study (GWAS) and biparental QTL mapping approaches to investigate the genetic architecture of coleoptile elongation in submerged rice plants. Genome-wide association studies are common in rice genetics research, with abundant allelic variations and molecular markers having been identified. Several published studies have demonstrated the utility of GWAS approaches in the detection of phenotype-genotype associations regarding various agronomic traits (Famoso et al. 2011; Huang et al. 2010; Zhao et al. 2011). It is noteworthy that the confounding over the deep population structure of rice and other self-fertilized plants (e.g., *Arabidopsis thaliana*) may result in missing heritability in GWAS results (Brachi et al. 2011). Re-structured mapping populations from biparental or multiparental intercrosses have compensated for the limitations of GWAS approaches (Brachi et al. 2010; Famoso et al. 2011). We compared the mapping results using two approaches and examined the factors that may contribute to the discrepancy of QTLs being detected. The QTLs identified in this investigation require further study, but may eventually serve as targets for breeding rice cultivars capable of germinating in anaerobic conditions.

## Results

### Variability of Anaerobic Germination in Two Mapping Populations

**Fig. 1:**
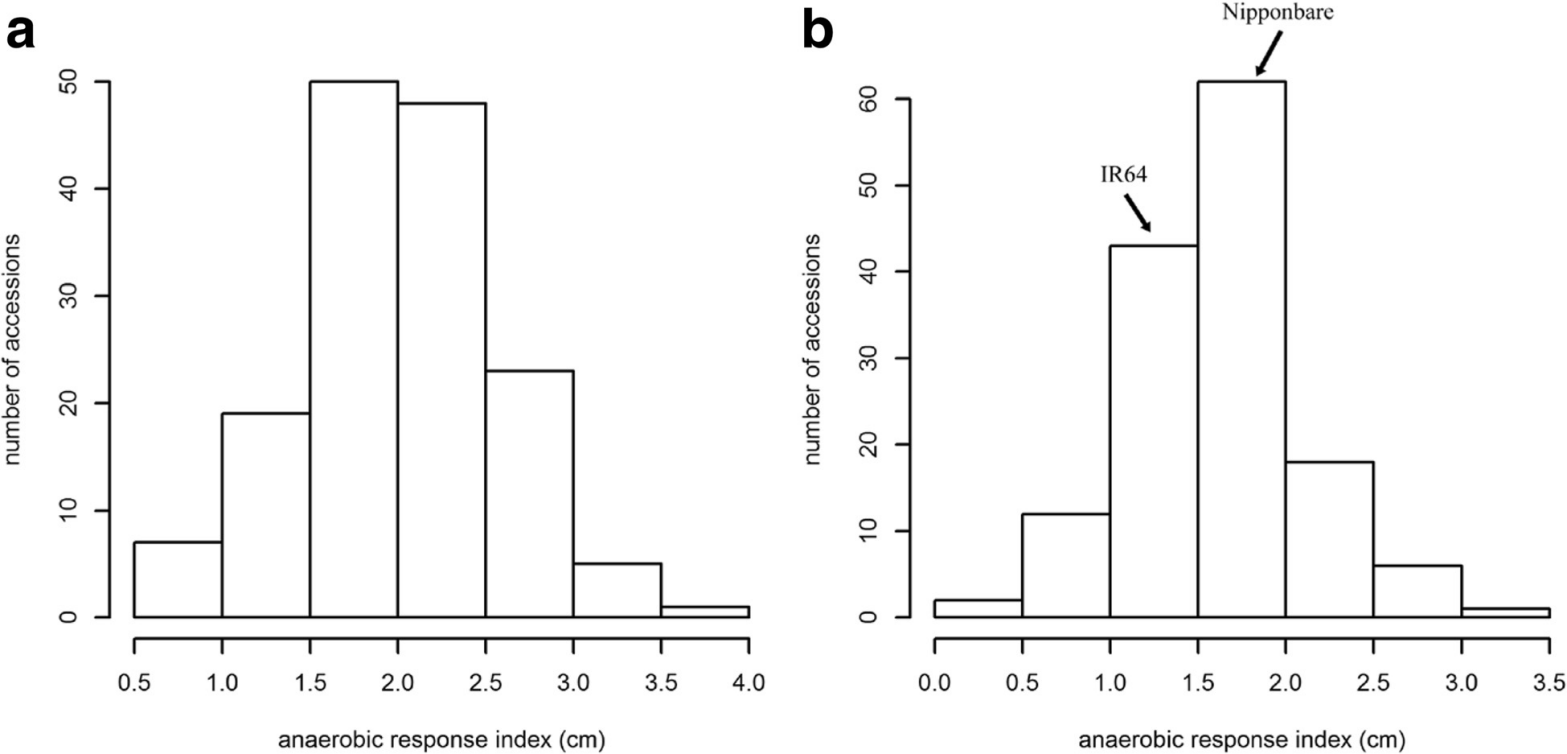
Variations in rice coleoptile growth in response to submerged conditions. Distribution of the anaerobic response index across 153 diverse accessions (*left*) and 144 biparental recombinant inbred lines (*right)*

The coleoptile lengths of plants from two mapping populations grown under normal conditions (control) or submerged in 5 cm of water are provided in Additional file 1: Table S1. The analysis of variance (ANOVA) of coleoptile growth for all genotypes indicated that the effects of treatment, genotype, and genotype × treatment were all significant (Additional file 2: Table S2). The difference in coleoptile length between control plants and those submerged in water for 7 days was designated the “anaerobic response index”, which represented the level of tolerance to anaerobic germination. Our results revealed that the anaerobic response index varied from 0.07 to 3.62 cm with a mean value of 1.84 cm among all phenotyped materials. The distribution of the anaerobic response index for each mapping population is provided in Fig. 1. In the biparental mapping population, the anaerobic response index of the parents, Nipponbare and IR64, were 2.08 and 1.36 cm, respectively. Transgressive segregation was observed in their F_10_ progenies, with the response index ranging from 0.07 to 3.04 cm (mean: 1.66 cm). The average response index (2.02 cm) and range (0.55–3.62 cm) of the global accessions were higher than those of the RIL population. When diverse accessions were grouped based on varietal characteristics, the *japonica* subgroup responded significantly better than the *indica* subgroup (*P* = 1.32E-06) (Additional file 3: Figure S1a). The *japonica* varieties had a mean response index of 2.18 cm and ranged from 0.64 to 3.62 cm, while the *indica* varieties had a mean response index of 1.66 cm and ranged from 0.55 to 2.54 cm. Twenty-seven accessions that did not belong to any subgroups were classified as *admix*. These accessions had a mean response index of 2.09 cm and ranged from 0.8 to 3.04 cm (Additional file 3: Figure S1a). We also observed significant variations in the ability to germinate anaerobically in five subpopulations: *tropical japonica* (TRJ), *temperate japonica* (TEJ), *indica* (IND), *aus* (AUS), and group V (ARO). Some *tropical japonica* accessions exhibited strong anaerobic germination (Additional file 3: Figure S1b).

### Genome-Wide Association Studies in Diverse Accessions

**Fig. 2:**
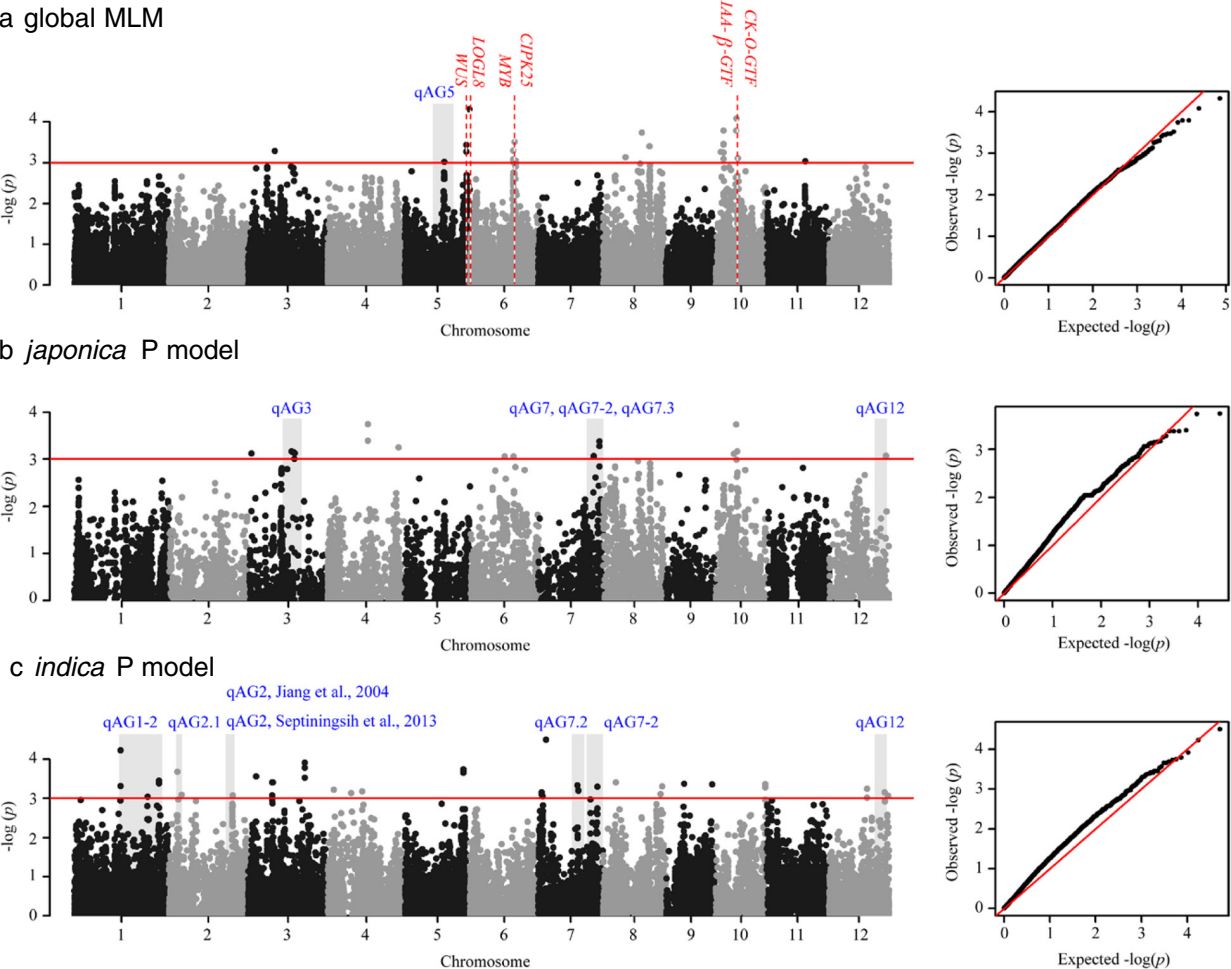
Manhattan plot of the SNPs associated with anaerobic germination. The red horizontal line indicates the significance threshold of multiple comparisons (*P* < 0.001). Genomic regions detected by our GWAS and previous biparental mapping studies are labeled with published QTLs in blue text. Candidate genes near the significant SNPs are indicated with a red vertical dashed line. The Q-Q plot corresponds to the fitness of the model

To identify genomic regions associated with anaerobic germination, we conducted a GWAS using 153 diverse accessions with a genotype dataset consisting of 36,901 single nucleotide polymorphisms (SNPs) (Zhao et al. 2011) and the anaerobic response index as the phenotype. Numerous significant SNPs that were detected using a general linear model (GLM) were false positives (Global GLM in Additional file 4: Figure S2). A mixed linear model (MLM) was used to control the confounding effect of the subpopulation structure and relatedness in the population (global MLM in Fig. 2). The results for the *japonica* and *indica* group-specific GWAS indicated that the confounding effect under the GLM was not severe (*japonica* GLM and *indica* GLM in Additional file 4: Figure S2). However, an overcorrection for the population structure was observed when both subpopulation structure and kinship matrix were considered in the mixed model (*japonica* MLM and *indica* MLM in Additional file 4: Figure S2). To address this overcorrection and avoid false negative results, a P model, which is a linear model that considers the first principal component (PC), was used to individually analyze the *indica* and *japonica* varietal groups. Only two main subpopulations were observed within each varietal group. We identified 88 significant SNPs as being associated with anaerobic germination in three analyses (*P* < 0.001) (Fig. 2 and Additional file 5: Table S3).

Significant SNPs detected in this study were compared with previously reported QTL intervals controlling shoot elongation (Jiang et al. 2004; Jiang et al. 2006) or survival rate (Angaji et al. 2010; Baltazar et al. 2014; Septiningsih et al. 2013) in submerged plants. We determined that ten genomic regions were co-localized (gray blocks in Fig. 2). In our GWAS of all accessions (Fig. 2a), one SNP (id5007272, *P* value = 9.61E-04) on chromosome five co-localized with *qAG5*, which is a QTL controlling submerged shoot elongation (Jiang et al. 2004) according to the physical position of the flanking markers. At the end of chromosome 7 (22.08–29.66 Mb) where multiple reported QTLs clustered (Angaji et al. 2010; Jiang et al. 2004; Septiningsih et al. 2013), four adjacent SNPs around 27.72 Mb and one SNP at 25.12 Mb were identified in the *japonica* varietal group (Fig. 2b). We detected another significant SNP at 26.92 Mb on chromosome seven within the *indica* subgroup. Additionally, within a large reported QTL interval (*qAG1–2*; (Angaji et al. 2010)), three independent SNP clusters were identified in the *indica* varietal group using the P model (Fig. 2c), highlighting the better resolution in the GWAS. Several genomic regions identified in this study, mostly in the varietal group-specific GWAS, were discovered in previous linkage mapping studies. These results confirmed the utility of GWAS approaches and also validated our experimental design.

In addition to known QTLs, we detected several new genomic regions. Based on the decay of linkage disequilibrium (LD) around significant SNPs and functional annotation of the genes in these regions, several candidate genes that may modulate the anaerobic response in rice were investigated. The most significant SNP (id5014906, *P* value = 4.82E-05) detected across all accessions was at 29.47 Mb on chromosome 5 (Fig. 2a). The LD between this SNP and its flanking markers decayed to R^2^ < 0.1 in an approximately 120 kb region (Additional file 6: Figure S3a) where 23 annotated genes were found. In this interval, there was a candidate gene encoding a cytokinin riboside 5’-monophosphate phosphoribohydrolase (*LOC_Os05g51390*, *LONELY GUY-like*, and *LOGL8*), which is an important cytokinin-activating enzyme. Previous functional studies demonstrated that the members of the *LOG* gene family regulate cytokinin activity during normal growth. For example, they influence the activity of shoot apical meristem in rice (Kurakawa et al. 2007) and alter root or shoot morphology in *A. thaliana* (Kuroha et al. 2009). Two other significant SNPs (id5013799 and id5013846) were detected at the end of chromosome five approximately 1.4 Mb away from the most significant SNP (id5014906). We observed a strong LD between these three SNPs (R^2^ > 0.6). Additionally, a *WUSCHEL* (*WUS*)-related homeobox gene (*WOX12*, *LOC_Os05g48990*) was located between the two significant SNPs (id5013799 and id5013846) (Additional file 6: Figure S3b). The *WUS* homeobox genes were reported to function in a cytokinin-related pathway to establish and maintain shoot meristems in *A. thaliana* (Leibfried et al. 2005; Mayer et al. 1998). On chromosome ten, we identified a highly significant SNP at 9.55 Mb (ud10000588, *P* value = 8.27E-05) across all accessions and another significant SNP (id10002607, *P* value = 1.63E-04) 170 kb farther away (Fig. 2a). A detailed LD analysis demonstrated that there was no strong correlation between a SNP (ud10000588) and its flanking markers (Additional file 6: Figure S3c). Similarly, the LD associated with another SNP (id10002607) decreased dramatically. However, a moderate LD (R^2^ = 0.45) was observed between the two single SNPs (Additional file 6: Figure S3c), suggesting that the significant effect of both markers was due to the same causal variant. In the region near the significant SNPs (id10002607 and ud10000588), two potential candidate genes were identified. One encoded an indole-3-acetate β-glucosyltransferase (*IAA* β*-GTF*, *LOC_Os10g18480*) and the other encoded a cytokinin-O-glucosyltransferase (*CK-O-GTF*, *LOC_Os10g18530*). Other anaerobic germination-related candidates, such as the MYB transcription factor (*LOC_Os06g35140*) and CBL-interacting protein kinase 25 (*CIPK25, LOC_Os06g35160*), were identified around a significant SNP (id6010625, *P* value = 3.06E-04) on chromosome 6 detected in a global MLM (Figs. 2a and Additional file 6: Figure S3d).

### QTL Mapping in a Biparental RIL Population

**Fig. 3:**
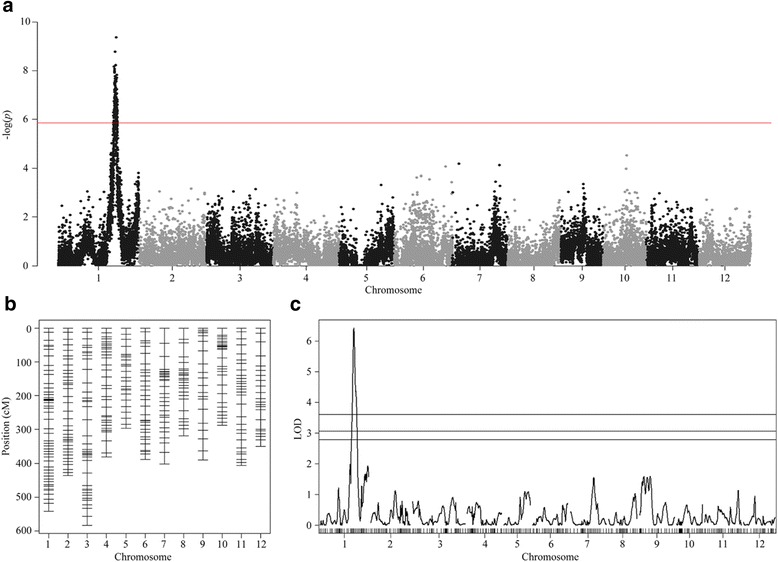
Biparental QTL mapping. **a** Single marker analysis. The red horizontal line indicates the significance threshold after Bonferroni’s adjustment. **b** Linkage map of 355 SNP markers. **c** QTL detection by interval mapping. Horizontal lines indicate the significant logarithm of odds threshold at 90, 95, and 99 % confidence levels (*from the bottom to the top*) based on 1000 permutations

We used 144 RILs derived from the Nipponbare/IR64 cross for mapping QTLs involved in anaerobic germination. We first used single marker analysis to test the association of 35,501 SNPs with variations in the anaerobic response index. After Bonferroni’s correction for all SNP markers with α = 0.05 (*P* value < 1.41 × 10^−6^), only one cluster containing 157 significant SNPs between 29.61 and 31.71 Mb on chromosome 1 was detected. The peak marker was at 31.0 Mb (Fig. 3a and Table 1). We also performed interval mapping using a linkage map generated from 355 SNPs (Fig. 3b). Only one QTL around 29–33 Mb on chromosome 1 was detected (Fig. 3c, Table 1). This region overlapped with the SNP clusters detected during single marker analysis. This interval was also located between the two peak markers identified in a previously reported anaerobic germination-associated QTL (*qAG1-2*, 26.04–32.03 Mb) (Angaji et al. 2010). We further examined the effect of the most significant SNP (S1_31006962) and observed that the *japonica* allele contributed to the increasing effect, which was consistent with a published mapping result (Angaji et al. 2010). This SNP explained approximately 27 % of the phenotypic variation in our RIL population.

**Table 1:** Major QTLs associated with anaerobic germination detected in the Nipponbare/IR64 RILs population

Chr.	Single marker analysis			Interval mapping (IM)			
	Peak marker	*P*-value	R^2^ (%)	Flanking markers	Peak marker	LOD	R^2^ (%)
1	S1_31006962	4.22E-10	26.38	S1_29187939	S1_30689538	6.42	18.56
				S1_32847451	S1_31361816		

**Fig. 4:**
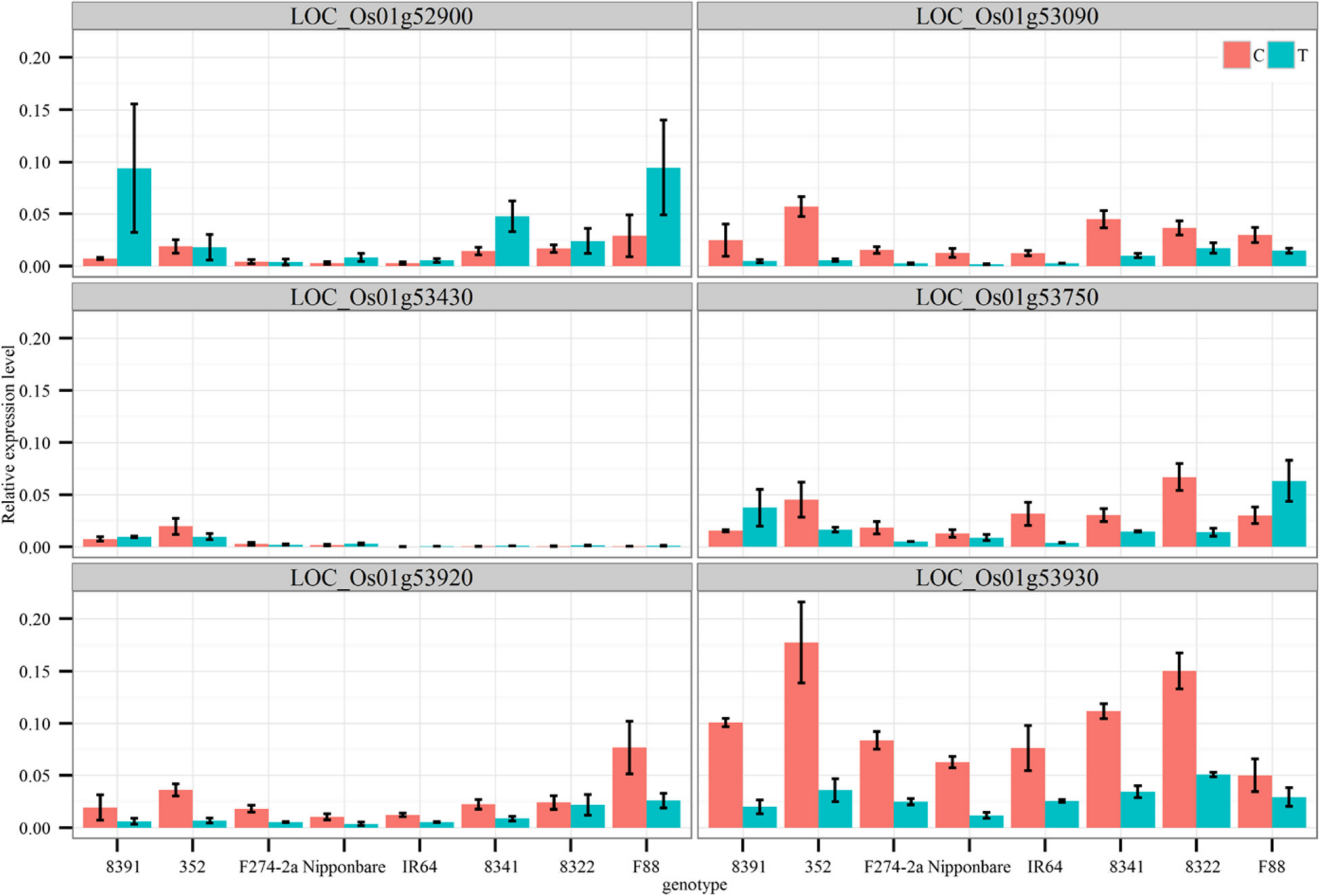
Expression analysis of candidate genes. The expression levels of six candidate genes in 4-day-old rice coleoptiles were measured using quantitative RT-PCR. The x-axis lists the eight genotypes with contrasting phenotypic performances (tolerant lines carrying *japonica* alleles: 8391, 352, F274-2a, and Nipponbare; sensitive lines carrying *indica* alleles: IR64, 8341, 8322, and F88). The y-axis indicates the expression levels relative to that of *OsACT1*. The red and blue boxes represent the control and submerged samples, respectively. The error bars correspond to the standard error (*n* = 3)

Within the significant QTL interval (29.61–31.71 Mb) on chromosome one, there were 13 differentially expressed genes in response to anoxic conditions in Nipponbare rice according to the results of a genome-wide transcriptome profile of 4-day-old anoxic rice coleoptiles (Lasanthi-Kudahettige et al. 2007). Among these anoxia-responsive genes, we examined the expression patterns of six candidate genes in diverse genotypes, including *LOC_Os01g53920*, which contains the most significant SNP (S1_31006962). The reverse transcription polymerase chain reaction (RT-PCR) was performed using total RNA isolated from 4-day-old coleoptiles of eight genotypes with contrasting responses to anaerobic conditions. Two of the six genes exhibited a consistently decreased expression level in submerged plants for all eight genotypes (Fig. 4). One of the genes encoded a putative infection-related protein (*LOC_Os01g53090*), while another encoded a hexokinase (*HXK6, LOC_Os01g53930*). Relative to the expression levels of control samples, the decrease in *HXK6* expression in submerged coleoptiles in *japonica* varieties (Nipponbare, 8391, and 352) was greater than that in *indica* varieties (IR64, 8341, and 8322). Hexokinases have been suggested to function as glucose sensors (Cho et al. 2006; Cho et al. 2009; Granot et al. 2014; Granot et al. 2013) that modulate the mechanisms associated with hypoxia and sugar starvation (Lim et al. 2013; Yim et al. 2012). Based on the evidence, *HXK6* may be a major factor affecting the phenotypic variations observed in the Nipponbare/IR64-derived RIL population.

### Haplotype Analysis of Candidate QTL Region in Diverse Germplasm

**Fig. 5:**
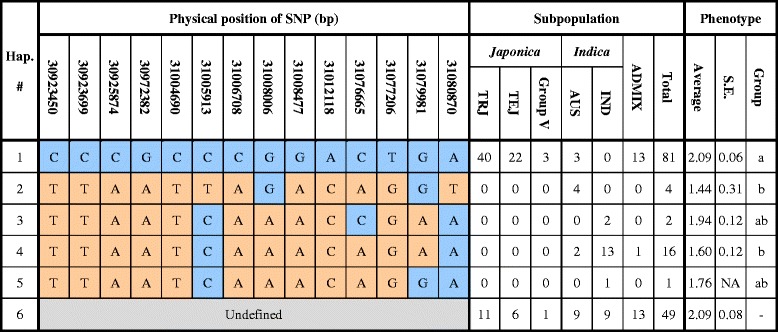
Haplotype analysis of candidate QTL in diverse accessions. Haplotypes observed across the 153 accessions using SNPs from a published data set. Five haplotypes were constructed according to 14 SNPs within the 158-kb region (30.923–31.081 Mb) flanking *HXK6*. There were 49 accessions with ambiguous genotypes, which were grouped into an undefined group. Physical positions of SNPs were based on IRGSP1.0 annotation and subpopulations were defined by principal component analysis. The Fisher’s least significance difference test with Bonferroni’s adjustment was used for multiple comparisons of phenotypic performance

The QTL region on chromosome 1 detected during biparental mapping was not significantly detected in our GWAS. To investigate whether the polymorphism in this region affected our results, we analyzed the allelic variation around the *HXK6* gene in 153 diverse accessions. Based on the original published SNP datasets and local LD structures, five haplotypes were observed in the approximately 158-kb region surrounding *HXK6* (Fig. 5). We observed a strong correlation between the subpopulation groups and haplotypes. Three *aus* varieties were grouped with *japonica*-specific haplotype 1, and none of the *japonica* rice varieties were observed to carry *indica*-specific haplotypes. The Fisher’s least significant difference test revealed that the anaerobic germination of the accessions carrying haplotype 1 was significantly better than that for the *indica* varieties carrying haplotype two or haplotype four. This finding implied that allelic variations within this 158-kb interval between *japonica* and *indica* rice contributes to the phenotypic variation of coleoptile responses to anoxic conditions.

## Discussion

### Coleoptile Elongation as an Escape Strategy to Tolerate Hypoxic Conditions During Germination

Seedling survival in flooded soil is an important trait for the success of a direct-seeding system. To identify suitable donor germplasm for breeding of rice varieties able to germinate anaerobically, more than 8000 accessions from the IRRI gene bank were screened for flooding tolerance during germination in plants submerged for 3 weeks (Angaji et al. 2010). Surprisingly, only 19 lines exhibited a survival rate over 70 %. Highly tolerant genotypes such as Khao Hlan On (Angaji et al. 2010), Ma-Zhan Red (Septiningsih et al. 2013), and Nanhi (Baltazar et al. 2014) have been selected to map QTLs associated with anaerobic germination. All of these studies assessed seedling survival 21 days after sowing. Very few QTLs associated with anaerobic germination have been identified, which is partly because of the phenotyping strategies and experimental conditions used.

In this study, we measured coleoptile length and used it to represent the ability to germinate anaerobically. The variability of coleoptile length was normally distributed in diverse accessions and RIL populations, which suggested its quantitative nature in submerged plants. Coleoptiles of *japonica* varieties tended to elongate faster than those of *indica* varieties. Accessions belonging to the *tropical japonica* subpopulation were particularly well adapted to germination in submerged conditions. The superior performance of the *japonica* varieties used as the parental line in interspecific crosses (this study; (Angaji et al. 2010; Jiang et al. 2004; Septiningsih et al. 2013)) demonstrated that the *japonica* alleles enhance anaerobic germination in segregating populations. Transgressive segregation observed in these biparental cross-derived populations suggest that epistatic interactions between the *japonica* and *indica* genomes significantly affect survival rate and coleoptile growth.

### Comparing GWAS and Biparental QTL Mapping Results

We identified several anaerobic germination-associated loci based on a GWAS. Some were novel discoveries and some co-localized with genomic intervals reported in previous biparental QTL studies. Multiple intervals at the end of chromosome seven have been associated with seedling survival or shoot elongation (Angaji et al. 2010; Jiang et al. 2004; Septiningsih et al. 2013). However, it is unclear whether a single locus or multiple loci located within this interval contributed to the effect. In our study, three genomic regions around 25.12, 26.92, and 27.72 Mb on chromosome 7 were detected in a varietal group-specific GWAS, suggesting that multiple allelic variations exist in the previously reported QTLs. The resolution of biparental mapping was insufficient to discriminate them from each other. The major QTL on chromosome 1 detected in our biparental mapping and the previously reported large-effect QTL on chromosome 9 (*qAG-9-2*; (Angaji et al. 2010)) were not mapped in our GWAS. Haplotype analyses eliminated the possibility that the QTL on chromosome 1 was a rare variant. When dealing with agronomic traits deeply associated with subpopulation structure, such as anaerobic germination, false positives are common in the absence of appropriate controls for the subpopulation and individual relatedness in GWAS analyses (Kang et al. 2008; Yu et al. 2005; Zhao et al. 2007). However, overcorrection of these potential confounding factors may produce false negative errors (Brachi et al. 2011; Korte & Farlow 2013). This possibility has been discussed in many studies investigating flowering time in *A. thaliana* (Brachi et al. 2010), plant height (Zhao et al. 2011), and aluminum tolerance in rice (Famoso et al. 2011). We observed strong correlations between haplotypes and varietal groups in the QTL interval on chromosome 1, which might explain why we were unable to detect them during our GWAS.

### Candidate Genes in Genomic Regions Detected in Two Mapping Studies

There is considerable evidence suggesting that *HXK6* may be involved in mediating the differences in coleoptile growth between *japonica* and *indica* rice varieties. First, only one chromosomal region carrying *HXK6* was detected in our *indica-japonica* RILs. This significant QTL co-localized with a peak interval containing *qAG1-2* (Angaji et al. 2010).

Second, cellular localization studies determined that HXK6 is a dual-targeted mitochondrial and nuclear protein (Cho et al. 2009; Huang et al. 2009), suggesting that HXK6 acts as a glucose sensor (Cho et al. 2006; Cho et al. 2009; Granot et al. 2014; Granot et al. 2013). Its involvement in hexose phosphorylation may modulate the expression of important hypoxia-inducible genes such as those for CIPK15 and alcohol dehydrogenase (Lim et al. 2013; Shingaki- Wells et al. 2014; Yim et al. 2012). Third, our RT-PCR results showed differential reductions in *HXK6* expression between tolerant and intolerant genotypes in 4-day-old coleoptiles submerged in water when compared with gene expression levels of the control group (Fig. 4). The decrease in *HXK6* expression in tolerant genotypes such as Nipponbare (5.3-fold) was greater than that in intolerant genotypes such as IR64 (3.0-fold). These differences were correlated with differences in coleoptile elongation (2.08 cm in Nipponbare and 1.36 cm in IR64). A previous study (Ismail et al. 2009) revealed that germinating seeds of tolerant varieties had lower starch concentrations and slightly higher soluble sugar levels than intolerant varieties during the first 8 days of flooding. Additionally, tolerant genotypes had higher amylase activity than intolerant genotypes in flooded conditions. Further studies examining the time-course and tissue-specific expression of *HXK6* in submerged plants and how the gene interacts with *RAmy3D*, *OsTPP7* and other anaerobic germination-related genes to promote coleoptile elongation are required to clarify the role of HXK6.

Ethylene is an important signaling phytohormone that quickly accumulates in submerged plants. Additionally, it stimulates multiple downstream mechanisms (Fukao & Bailey-Serres 2008). Fine mapping of *Sub1* and *SNORKEL* confirmed that ethylene response factors affect rice responses to submerged conditions (Fukao et al. 2006; Hattori et al. 2009; Xu et al. 2006). Interestingly, we did not identify any genes related to ethylene signaling or ethylene biosynthesis in the two mapping studies. In contrast, in the genomic regions highly associated with anaerobic germination, there were genes affecting cytokinin and auxin activities (chromosomes 5 and 10). A previous study demonstrated that the auxin-gibberellin interaction promotes coleoptile elongation in rice (Kefford 1962). The analysis of many semi-aquatic eudicot species, such as *Regnellidium diphyllum*, has revealed that auxin is required for the submergence/ethylene response (Jackson 2008). A recent study on the *Sub1A*-mediated submergence responsive expression profiles reported that several genes involved in cytokinin-mediated delay of senescence in leaves were significantly enriched in the dataset (Jung et al. 2010). We observed a similar delayed senescence in our submerged coleoptiles. Two candidate genes identified in this study, *LOGL8* and *WUS*-related homeobox, are possibly involved in a cytokinin-related pathway to establish and maintain the activity of rice shoot apical meristem. Future experiments are needed to confirm how these phytohormones and their regulatory mechanisms contribute to the tolerance of submerged conditions during anaerobic germination.

In the region containing significant SNPs on chromosome six, we identified a gene encoding CIPK25, whose functions may be similar to those of homologous genes in response to hypoxia or energy deficit. Ca^2+^ signal transduction is involved in the detection of energy or oxygen deficits and the modulation of downstream mechanisms to promote anaerobic germination in rice (Lee et al. 2009). Calcineurin-interacting protein kinase is important for the detection of the Ca^2+^ signal and the subsequent stimulation of the downstream phosphor-signaling cascade. A mutant *cipk15* line showed poor anaerobic germination and seedling growth under submerged conditions (Lee et al. 2009). A CIPK15-SnRK1A-MYBS1 phosphorylation cascade promotes the expression of *RAmy3D* (Lee et al. 2009; Lu et al. 2002; Lu et al. 2007), which is an important enzyme highly expressed in response to oxygen/energy deficits to accelerate the mobilization of starch in the endosperm (Guglielminetti et al. 1995a, 1995b; Hwang et al. 1998; Hwang et al. 1999; Perata et al. 1997). In the same genomic region on chromosome six, another gene encoding a putative MYB transcription factor may promote anaerobic germination in rice. It was reported that in addition to MYBS1, there are other MYB transcription factors involved in the regulation of *RAmy3D* (Lu et al. 2002).

## Conclusions

In this study, we demonstrated the utility of combining GWAS and biparental QTL mapping approaches to identify the chromosomal regions controlling coleoptile elongation in rice plants submerged in water. The significant genomic regions including the QTL carrying *HXK6* are potential candidates that should be studied further and incorporated into elite rice cultivars to improve seedling survival during anaerobic germination.

## Methods

### Plant Materials

Two types of rice mapping populations were screened for anaerobic germination: (1) *Diverse germplasm*: 153 rice accessions, clustered into two major varietal groups, *indica* and *japonica*, were obtained from the United States Department of Agriculture Genetic Stocks-*Oryza* (USDA-GSOR) gene bank (Zhao et al. 2011); (2) *RILs*: 144 lines (F_10_) were derived from a cross between the *japonica* and *indica* cultivars, Nipponbare and IR64 (Yan et al. 2015). The seeds were dried in a heated air dryer at 37 °C for 5 days after harvest and then stored at 4 °C. Considering seed age affects anaerobic germination, we only used seeds that were about the same age (less than 18 months old). To avoid the effects of seed dormancy, the roll paper method was used to assess the ability of each accession to germinate, the details of the method can be found in http://www.knowledgebank.irri.org/step-by-step-production/pre-planting/seed-quality. Dormant seeds were excluded. The genotypic data for the two mapping populations were downloaded from http://ricediversity.org/data.

### Phenotyping for Anaerobic Germination

Seeds were surface sterilized in 20 % diluted bleach (6–7 % NaClO) for 20 min and then thoroughly rinsed with water. For control samples, ten sterilized seeds were placed on water-soaked Kimwipe tissues in capped 8-cm tall glass tubes. For submerged samples, the sterilized seeds were submerged in sterilized water (up to 5 cm in the 8-cm tube). The germination experiment was conducted in a growth chamber with a 16 h light (150 μmol m^−2^ s^−1^)/8 h dark cycle at 25 °C. After 7 days, coleoptile length was measured using a standard ruler and the anaerobic response index was calculated (submerged coleoptile length − control coleoptile length).

### Genome-Wide Association Mapping

For diverse accessions, two statistical methods were used to perform genome-wide association mapping. The first approach involved single marker analysis based on a GLM: *Y = βX + ε*, where *Y* and *X* are vectors of phenotype and genotype data, *β* represents the SNP effects and *ε* corresponds to random error. Using single marker analysis, we evaluated the effect of each SNP marker and identified the potential causal SNPs or chromosomal regions contributing to variation in the ability to germinate anaerobically. Our second approach considered the confounding effects of subpopulation differentiation and relatedness between individuals. To compensate for these confounding effects, the MLM was used: *Y* = *βX* + *γP* + *Zu* + *ε Y*, *X*, and *β* are as described above, *P* is a vector of the PC resolving population structure, *γ* is the effect of population structure, *u* refers to the random effect from kinship $u\sim N\left(0,{\sigma}_g^2K\right)$, Z is a coincident matrix, and *ε* corresponds to random error $\varepsilon \sim N\left(0,{\sigma}_e^2I\right)$ (Kang et al. 2008; Yu et al. 2005; Zhang et al. 2010). These analyses were completed using TASSEL 4.0 software (Bradbury et al. 2007) and R/GAPIT (Lipka et al. 2012).

### Estimation of Linkage Disequilibrium

The LD structure around the significant SNPs was estimated using TASSEL 4.0 software (Bradbury et al. 2007). When two markers had a low LD (R^2^ < 0.1), this interval was considered a candidate region where potential causal variants may reside. Gramene Mart (http://www.gramene.org/) was used to search for candidate genes in the target region.

### QTL Analysis

For 144 RILs, single marker regression based on a GLM was completed to examine the association between each SNP and the anaerobic response index. The raw *P* values were adjusted using a Bonferroni-corrected α value of 0.05 as a threshold to indicate the significance of the QTL interval. For linkage analyses, we selected 355 SNPs uniformly distributed from the original marker set to construct a linkage map using the Kosambi map function in R/qtl (Arends et al. 2010). Interval mapping was used to identify QTLs. Thresholds to determine the significance of QTLs were determined based on 1000 permutations.

### RNA Preparation and Quantitative RT-PCR

Total RNA from coleoptiles of 4-day-old seedlings was extracted using TRI Reagent® (Invitrogen, MA, USA) and the Direct-zol™ RNA MiniPrep kit (Zymo Research, CA, USA) following the manufacturer’s instructions. Gene-specific primers were designed using NCBI primer BLAST (http://www.ncbi.nlm.nih.gov/tools/primer-blast/). The primer sequences are listed in Additional file 7: Table S4. Quantitative RT-PCR was performed using the Rotor-Gene® SYBR® Green RT-PCR kit (Qiagen, Hilden, Germany) according to the relevant manufacturer’s protocol for one step RT-PCR. The PCR conditions were as follows: 10 min at 55 °C for reverse transcription, 5 min of pre-denaturation at 95 °C, 40 cycles of 5 s at 95 °C and 10 s at 60 °C, and melting curve generation. The *OsACT1* gene (forward primer: 5’-ATGAAGATCAAGGTGGTCGC-3’; reverse primer: 5’-GTACTCAGCCTTGGCAATCC-3’) was selected as an internal reference for relative quantification. The delta-delta C_T_ method was used to calculate the relative expression level fold-changes between submerged and control samples (Pfaffl 2001).

## Additional files

Additional file 1: Table S1. Phenotypic data of all samples. (XLSX 22 kb) Additional file 2: Table S2. Treatment effect and treatment-genotype interaction tested by ANOVA. (DOCX 17 kb) Additional file 3: Figure S1. Distribution of anaerobic response index in subgroups. (PPTX 21261 kb) Additional file 4: Figure S2. Manhattan plot from GWAS of anaerobic response index within and across subspecies. (PPTX 3009 kb) Additional file 5: Table S3.
*P*-value from GWAS results. (XLSX 7021 kb) Additional file 6: Figure S3. LD of the peak SNPs from GWAS. (PPTX 2487 kb) Additional file 7: Table S4. List of primers. (XLSX 11 kb)
