# Supplementary material for: Genetic Mapping of Anaerobic Germination-Associated QTLs Controlling Coleoptile Elongation in Rice
Source: Rice (N Y). 2015 Dec 23;8:38. doi: 10.1186/s12284-015-0072-3 (PMC4689725; doi:10.1186/s12284-015-0072-3)
Supplement: Additional file 2: Table S2. — Treatment effect and treatment-genotype interaction tested by ANOVA. (DOCX 17 kb) [file 12284_2015_72_MOESM2_ESM.docx]

Table S2. Treatment effect and treatment-genotype interaction by ANOVA

|  | Df | Sum of  square | Mean of square | F value | Pr(>F) |
| --- | --- | --- | --- | --- | --- |
| Treatment effect | 1 | 2343.66 | 2343.66 | 28737.8895 | ~0 |
| Treatment-genotype  interaction | 592 | 382.99 | 0.65 | 7.9329 | ~0 |
| Residuals | 2030 | 165.55 | 0.08 | NA | NA |
