# Supplementary figures and images for: Genetic Mapping of Anaerobic Germination-Associated QTLs Controlling Coleoptile Elongation in Rice
Source: Rice (N Y). 2015 Dec 23;8:38. doi: 10.1186/s12284-015-0072-3 (PMC4689725; doi:10.1186/s12284-015-0072-3)

## Slide 1
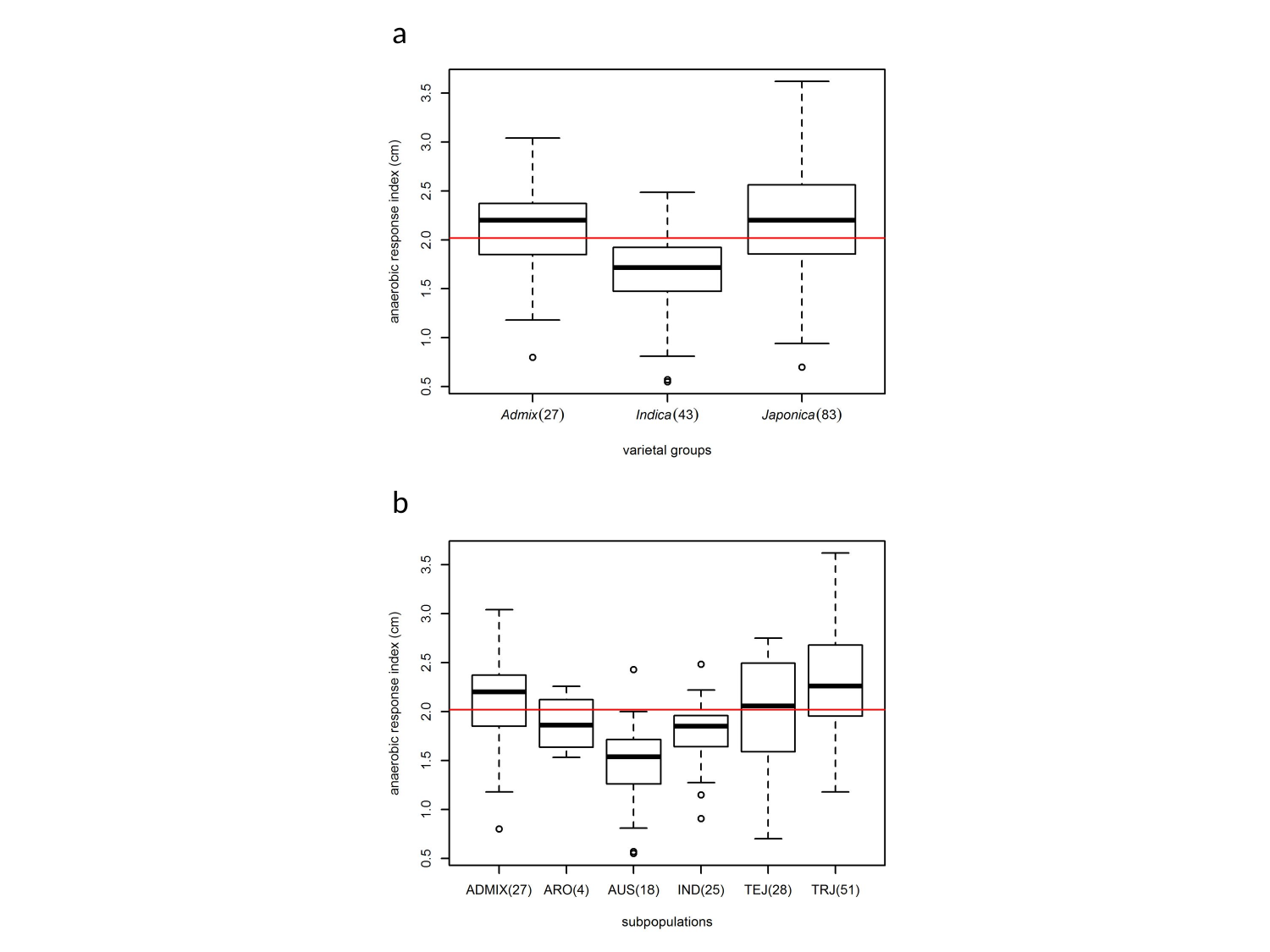

a
b

Supplement: Additional file 3: Figure S1. — Distribution of anaerobic response index in subgroups. (PPTX 21261 kb) [file 12284_2015_72_MOESM3_ESM.pptx]

## Slide 1
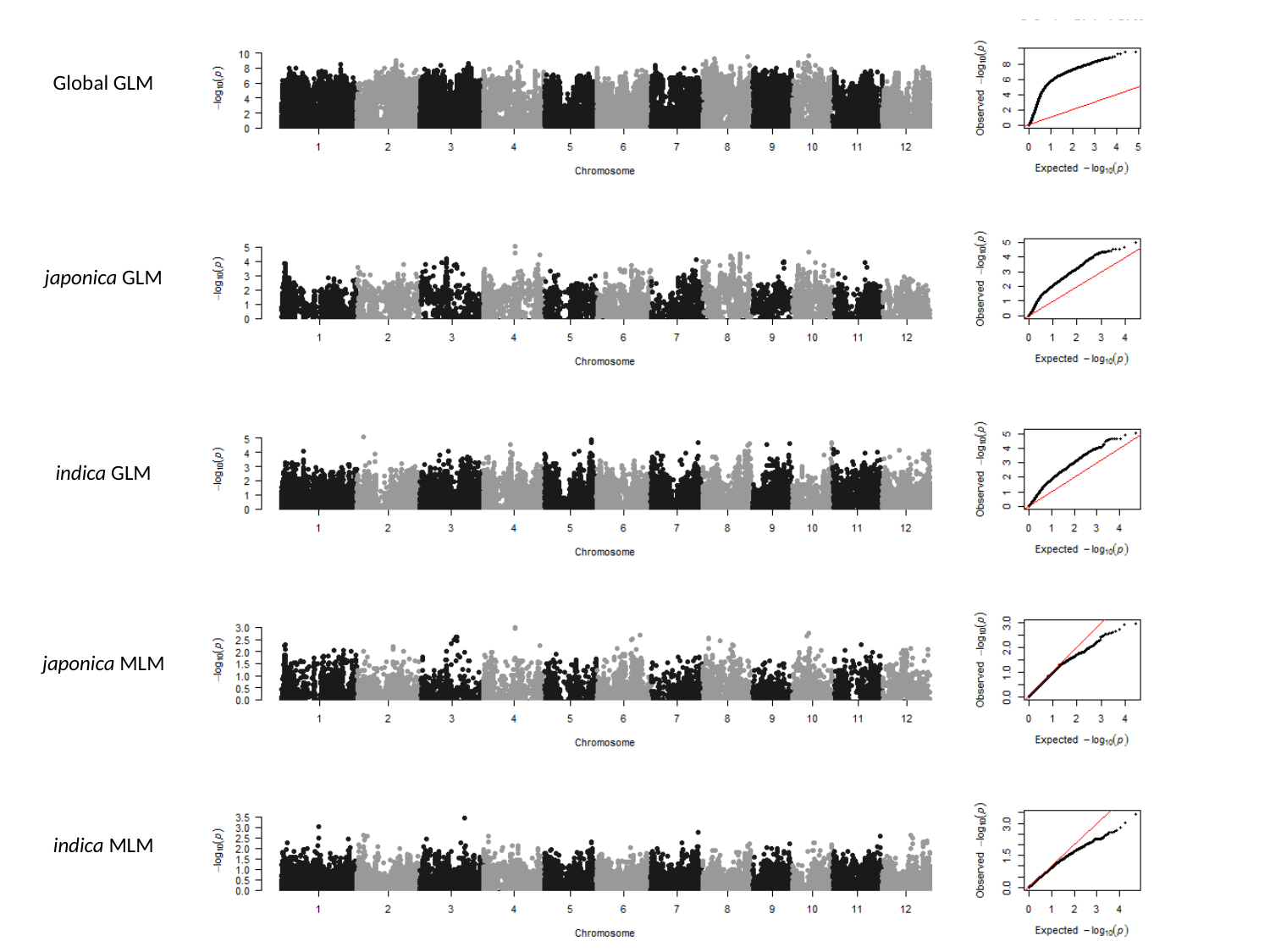

Global GLM
japonica GLM
indica GLM
japonica MLM
indica MLM

Supplement: Additional file 4: Figure S2. — Manhattan plot from GWAS of anaerobic response index within and across subspecies. (PPTX 3009 kb) [file 12284_2015_72_MOESM4_ESM.pptx]

## Slide 1
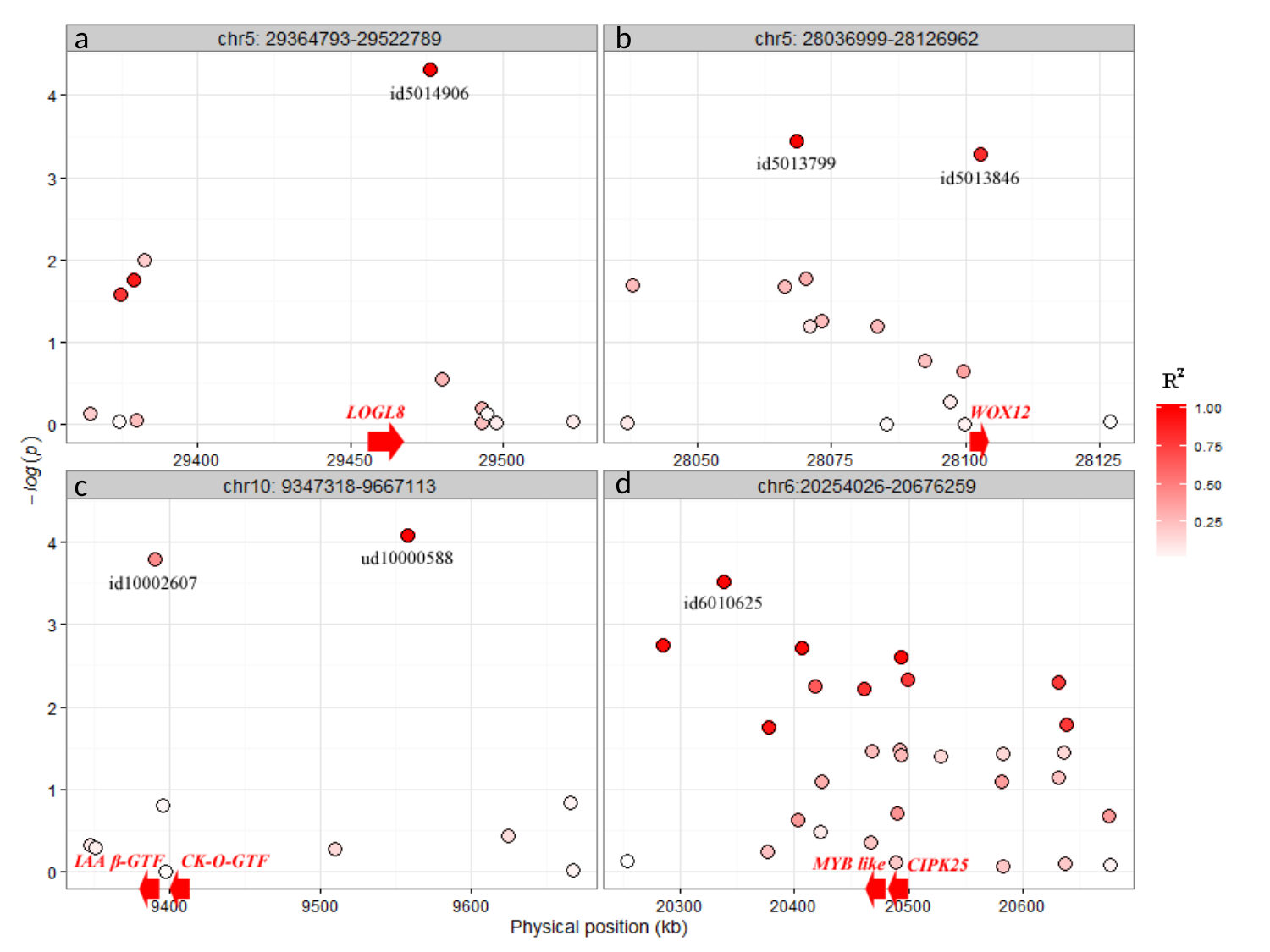

a
b
d
c

Supplement: Additional file 6: Figure S3. — LD of the peak SNPs from GWAS. (PPTX 2487 kb) [file 12284_2015_72_MOESM6_ESM.pptx]
